# Diagnostic Significance in Estimating Tumor Burden Using Extracellular Salivary Biomarkers in Gastric Cancer Patients

**DOI:** 10.3390/jcm14103596

**Published:** 2025-05-21

**Authors:** Sung Eun Oh, Jong Bae Seo, Jeongeun Noh, Sung Kim, Yong Kim, Ji Yeong An

**Affiliations:** 1 Department of Surgery, Samsung Medical Center, Sungkyunkwan University School of Medicine, Seoul 06351, Republic of Korea; ohsungeun228@gmail.com (S.E.O.); skim.kim@samsung.com (S.K.); 2 Department of Biosciences, Mokpo National University, Muan 58554, Republic of Korea; jbseo@mokpo.ac.kr; 3 Department of Biomedicine, Health & Life Convergence Sciences, BK21 Four, Biomedical and Healthcare Research Institute, Mokpo National University, Muan 58554, Republic of Korea; mm4n021j12@naver.com; 4 School of Dentistry, University of California, Los Angeles, CA 90095, USA; thadyk@ucla.edu

**Keywords:** salivary extracellular RNA, biomarker, gastric cancer, gastrectomy, liquid biopsy

## Abstract

**Background:** We investigated the possibility of predicting tumor burden with salivary extracellular RNA (exRNA) biomarkers in gastric cancer patients. **Methods:** Saliva samples were prospectively collected from 50 gastric cancer patients who underwent gastrectomy with curative intent. Approximately 5 mL of saliva was collected before surgery and on the 5th to 7th days after surgery. The expression of three mRNAs (SPINK7, PPL, and SEMA4B) and two miRNAs (miR140-5p and miR301a) that were previously validated was determined by reverse transcription quantitative real-time PCR. **Results:** There were significant differences in the pre-operative expression of PPL (*p* = 0.025), SEMA4B (*p* = 0.012), and miR140-5p (*p* = 0.036) between pathologic stage I/II and III/IV groups. The area under the curve (AUC) of each respective multivariable model in predicting stage III/IV, which was adjusted for age and sex, was 75.4% (PPL), 82.5% (SEMA4B), and 75.5% (miR140-5p). In the multivariable model, including all three biomarkers, the AUC was 89.2%. On the other hand, none of the conventional tumor markers (CEA, CA19-9, and CA72-4) could predict tumor burden before surgery. The AUC of the multivariable model, including CEA, CA19-9, and CA72-4, was 67.2%, 66.2%, and 67.4%, respectively. When all three tumor markers were included in the multivariable model, the AUC was 70.5%. **Conclusions:** Noninvasively detected salivary biomarkers have been shown to have higher diagnostic accuracy than conventional tumor markers detected by invasive blood tests for estimating pre-operative tumor burden. This study demonstrates the potential utility of these biomarkers in pre-operative risk assessment and monitoring surgical treatment response to gastric cancer.

## 1. Background

Gastric cancer is one of the most common cancers worldwide. It ranked fifth in incidence and was the fourth leading cause of death among all solid cancers, excluding non-melanoma skin cancer, globally in 2020 [1]. The Korean National Cancer Screening Program (KNCSP) for gastric cancer played a pivotal role in increasing the number of curable cancers by early detection, eventually improving overall survival [2]. However, upper endoscopy for GC detection in KNCSP is costly, time-consuming, and invasive. Therefore, there is a need for predictive biomarkers that can be used as a credible, noninvasive screening tool for early detection of gastric cancer.

Salivary extracellular RNA (exRNA) biomarkers, including miRNAs, have been established for detecting various local and systemic diseases, such as oral cancer [3] and Sjögren syndrome [4]. Notably, several studies have revealed that solid tumors such as pancreatic cancer [5], breast cancer [6], and lung cancer [7], which are distal from the oral cavity, produce discriminatory biomarkers in saliva.

A previous study on salivary exRNA biomarkers showed that three mRNAs (SPINK7, PPL, and SEMA4B mRNAs) and two miRNAs (miR140-5p and miR301a) were significantly down-regulated in gastric cancer patients compared with healthy individuals in a Korean population [8]. The positive predictive value and negative predictive value of the biomarkers were 82% and 77%, respectively, and the area under the curve (AUC) value of the receiver operating characteristic (ROC) curve was 0.81. When clinical information such as age, sex, and smoking was included, the AUC value increased to 0.87, which signified their feasibility as diagnostic markers of gastric cancer.

In this study, we prospectively collected saliva from 50 gastric cancer Korean patients and investigated the expression of the five biomarkers. We performed multivariate analysis with pre-operative expression levels to evaluate the potential of the biomarkers in predicting tumor burden and compared the performance with conventional tumor markers. In addition, we evaluated the expression of the biomarkers before and after curative gastrectomy to assess whether the markers reflected the surgical treatment response.

## 2. Methods

### 2.1. Patients and Study Design

This study was performed at Samsung Medical Center (Republic of Korea) and Mokpo National University (Republic of Korea) with approval from the Institutional Review Board of Samsung Medical Center (SMC IRB 2019-12-089). Patients diagnosed with gastric cancer were recruited from Samsung Medical Center, and saliva samples were prospectively collected before and after surgery. Inclusion criteria were as follows: age 19 years or older, diagnosis of gastric cancer, underwent radical gastrectomy, no pre-operative treatment (neoadjuvant or palliative chemotherapy or radiotherapy), written informed consent, and sufficient quantity and quality of collected saliva for analysis. We excluded patients younger than 19 years, those not diagnosed with gastric cancer, those receiving palliative surgery or pre-operative treatment, those who had distant metastasis or peritoneal seeding, and those who did not provide informed consent. Patients were also excluded if the quality or quantity of saliva was not sufficient for analysis.

Clinicopathologic characteristics, including age, sex, pre-operative levels of tumor markers (CEA, CA19-9, and CA72-4), *H. pylori* infection, histologic type, Lauren type, tumor size, and pathologic stage, were ascertained from medical records. *H. pylori* infection was confirmed with endoscopic biopsy or serum antibody test (IgG). Blood tests were performed 1–2 months prior to surgery to evaluate the pre-operative tumor markers. Tumor histology was dichotomized as differentiated, which included papillary adenocarcinoma and well or moderately differentiated adenocarcinoma, or undifferentiated, which included poorly or undifferentiated adenocarcinoma, signet ring cell carcinoma, mucinous carcinoma, and other types. Pathologic stage was classified according to the eighth edition of the American Joint Committee on Cancer Classification [9].

### 2.2. Collection and Storage of Samples

Saliva was collected before surgery and on the 5th to 7th days after surgery. Eating or drinking except water, smoking, and oral washing with gargle agents were prohibited at least 1 h before the collection. For saliva collection, the patients rinsed their mouths with drinking water, and approximately 5 mL of saliva was collected into the sample container. Saliva collection was performed using the spitting method as described previously [10]. Five milliliters of saliva were collected and centrifuged at 2000× *g* for 5 min at 24 °C. The cell-free supernatants were stored at −80 °C in a 1.8 mL cryotube until use.

### 2.3. Salivary exRNA Extraction

The total exRNA was extracted from 300 µL of saliva supernatant using the miRNeasy micro kit (Qiagen, Venlo, The Netherlands) following the manufacturer’s instructions. After mixing with QIAzol reagent to denature saliva samples, the samples were transferred and further processed on a designated RNA workbench in the lab. The quality of salivary exRNA was evaluated by detecting expression levels of a saliva internal reference gene using reverse transcription real-time polymerase chain reaction (RT-qPCR) [11]. The specificity of the PCR product for each gene was confirmed with melting curve analysis. We calculated ΔCq by subtracting the Cq value of the housekeeping gene from the raw Cq value of each biomarker.

### 2.4. RT-PCR of mRNA

The multiplex RT-PCR pre-amplification was performed with the SuperScript III Platinum One-Step qRT-PCR System (Invitrogen, Waltham, MA, USA) with a pool of outer primers at 100 nM each. The reaction mixture was prepared on ice and then loaded into the pre-heated thermocycler. The reaction conditions were as follows: 2 min at 60 °C, 30 min at 50 °C, 2 min at 95 °C, and 15 cycles of 15 s at 95 °C, 30 s at 50 °C, 10 s at 60 °C, and 10 s at 72 °C, with a final extension for 10 min at 72 °C and cooling to 4 °C. Immediately after RT-PCR, 10 μL of the reaction were treated with 4 μL of Exo-SAP-IT (Applied Biosystems, Foster City, CA, USA) for 15 min at 37 °C to remove excess primers and dNTPs, and the mixture was heated to 80 °C for 15 min to inactivate the enzyme mix. The pre-amplified cDNAs were then diluted (20-fold) by adding water to 200 μL.

Singleplex qPCR was performed in a 10 µL reaction with 2 µL of each pre-amplified cDNA sample and the inner primers at 200 nM each. The reaction was conducted with SYBR Green I Master mix using the CFX connect (BioRad, Hercules, CA, USA) instrument. After 10 min of polymerase activation at 95 °C, 40 cycles of 15 s at 95 °C and 60 s at 60 °C were performed, followed by melting curve analysis. Three controls, RT-control, no-template control, and positive control with universal human RNA, were performed with every biomarker on each sample.

### 2.5. RT-PCR of miRNA

Total exRNA (3 ng) was converted to complementary DNA using the TaqMan miRNA Reverse Transcription Kit (Applied Biosystems) according to the manufacturer’s instructions. The RT product was preamplified using TaqMan PreAmp Master Mix (Applied Biosystems) and the TaqMan microRNA assay (Applied Biosystems). The product was not diluted before miRNA quantification. The resulting cDNA was used as a template for real-time quantitative PCR analysis in a total reaction volume of 20 μL. All reactions were performed in triplicate using TaqMan microRNA Assays (Applied Biosystems, Cat. No. 4427975) specific for miR140-5p (Assay ID: 001187) and miR301a-3p (Assay ID: 000528). Each probe was labeled with a 5′ FAM fluorophore and a 3′ non-fluorescent quencher (NFQ) with a minor groove binder (MGB). ΔCq values were calculated using RNA polymerase III–transcribed U6 small nuclear RNA (Assay ID: 001973) and miR197 (Assay ID: 000497) as reference genes.

### 2.6. Statistical Analysis

To calculate the expression, the formula ΔCq = Cq(selected m/miRNA) − Cq(internal validation) was used, where ΔCq was defined as the difference in quantification cycle (Cq) values. Each sample was analyzed in triplicate and repeated three times. When there were more than two outliers among the three samples, the mean expression level was not included in this analysis.

Statistical differences in mRNA or miRNA expression levels in gastric cancer patients were analyzed by an independent *t*-test or one-way ANOVA test, and the differences between pre-operative and post-operative levels were tested using a paired *t*-test. To evaluate the differences in changes between subgroups, we used an independent *t*-test or a one-way ANOVA test. A multivariable model, including clinical characteristics and biomarkers, was analyzed using logistic regression to predict advanced disease (pathologic stage III or higher). In this model, Firth’s penalized likelihood approach was applied because of the small sample size. Furthermore, receiver operating characteristic (ROC) curve analysis was conducted to evaluate the diagnostic value of mRNA or miRNA and the clinical characteristics in differentiating between pathologic stage I/II and stage III/IV. We used Youden’s index to determine the optimal cut-off value to evaluate the probability, sensitivity, and specificity of each multivariable model. SAS version 9.4 (SAS Institute, Cary, NC, USA) and R 4.0.4 (Vienna, Austria; http://www.R-project.org/, accessed on 1 July 2023) were used for statistical analysis. Differences with a *p* < 0.05 were considered statistically significant.

## 3. Results

This study included 50 patients with gastric cancer. The general characteristics and clinicopathological factors of the patients are provided in Table 1. Approximately 66% of patients were male; 23 (46%) were diagnosed with stage III, and three (6%) were diagnosed with stage IV. Lymphatic invasion was detected in 62% (31/50) of patients.

**Figure 1:**
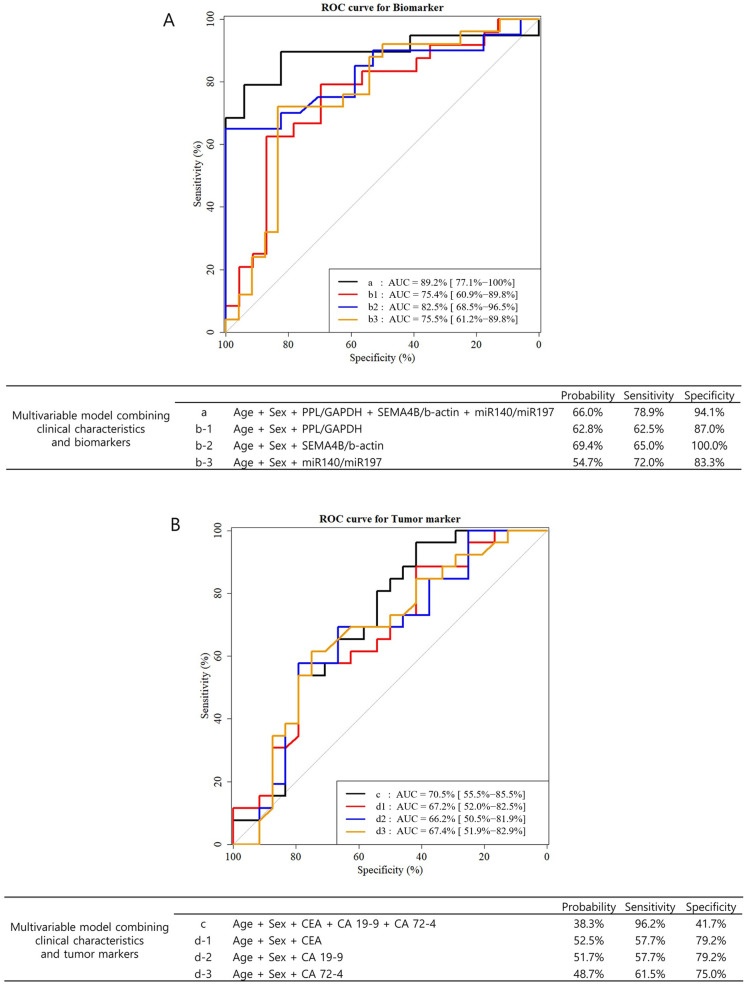
The receiver operator characteristic (ROC) curves and area under the curve (AUC) of multivariable models of pre-operative biomarkers and tumor markers in predicting tumor burden (stages III and IV). A multivariable model was analyzed using logistic regression. In this model, Firth’s penalized likelihood approach was applied because of the small sample size. (**A**) The ROC curves of multivariable models combining clinical characteristics (age and sex) and three biomarkers (PPL, SEMA4B, and miR140-5p). (**B**) The ROC curves of multivariable models combining clinical characteristics (age and sex) and three conventional tumor markers (CEA, CA19-9, CA72-4).

The pre-operative expression levels of tumor markers (CEA, CA19-9, and CA72-4) and salivary extracellular RNA biomarkers in patient subgroups are shown in Table 2. There were significant differences between stages I/II and III/IV patients in pre-operative expression of PPL/GAPDH (−6.2 ± 2.4 vs. −7.9 ± 2.4; *p* = 0.025), SEMA4B/ACTB (10.5 ± 0.9 vs. 11.9 ± 1.6; *p* = 0.012), and miR140-5p/miR197 (−1.3 ± 0.8 vs. −0.8 ± 0.7; *p* = 0.036). However, there were no significant differences between the compared groups in pre-operative expression of tumor markers. Univariable and multivariable analyses were performed with the three biomarkers to investigate the potential of discriminating between stage I/II and stage III/IV patients before surgery by pre-operative levels of biomarkers and clinicopathologic factors. In the multivariable model adjusted for age and sex, the three biomarkers were statistically significant factors (Table 3). The AUC of the multivariable model, including PPL/GAPDH, SEMA4B/ACTB, and miR140-5p/miR197, was 75.4%, 82.5%, and 75.5%, respectively (Figure 1A). In the multivariable model, including all three biomarkers, the AUC was 89.2%. In a multivariable model adjusted for age, sex, *H. pylori* infection, histologic type, Lauren type, and tumor size, PPL/GAPDH (odds ratio 0.66 per 1 unit; *p* = 0.041) was the only statistically significant biomarker (Table S1). The AUC of the multivariable model, including PPL/GAPDH, SEMA4B/ACTB, and miR140-5p/miR197, was 84.1%, 87.9%, and 81.7%, respectively (Figure S1). In the multivariable model with all three markers, the AUC was 92.0%. For comparison, we also performed univariable and multivariable analyses using conventional tumor markers (CEA, CA19-9, and CA72-4), none of which could predict tumor burden before surgery (Table 4). The AUCs of the multivariable model, including CEA, CA19-9, and CA72-4, were 67.2%, 66.2%, and 67.4%, respectively (Figure 1B). When all three tumor markers were included in the multivariable model, the AUC was 70.5%.

Saliva samples were obtained from gastric cancer patients before and after surgery and analyzed. Table 5 shows the relative expression levels of salivary biomarkers before and after gastrectomy. Among the five biomarkers, only SPINK7 showed a decrease after gastrectomy, but the difference was not statistically significant. A significant increase was observed in the mean values of miR140-5p/miR197 (0.4 ± 0.9, *p* = 0.002) and miR301a/miR197 (0.7 ± 1.5, *p* = 0.006) five days after surgery. In subgroup analysis (Table S2), a significant increase after surgery in the mean values of miR140-5p/miR197 was observed in old (≥60 years) and male patients. In contrast, the mean value of miR301a/miR197 was significantly increased in young (<60 years) and female patients. Both miR140-5p/miR197 and miR301a/miR197 significantly increased post-operatively in the differentiated histologic type group and intestinal Lauren type group (all variables, *p* < 0.05).

In advanced gastric cancer (pathologic stages III and IV) patients, the post-operative expression levels of PPL/GAPDH, miR140-5p/miR197, and miR301a/miR197 were significantly increased compared with the pre-operative levels (Table 6). The increase in post-operative level was highest for PPL/GAPDH (pre-operative −7.9 ± 2.4, post-operative −6.1 ± 2.5, difference 1.8 ± 3.2, *p* = 0.019).

## 4. Discussion

Among the known salivary extracellular RNA biomarkers examined in this study, the pre-operative expression levels of PPL, SEMA4B, and miR140-5p were significantly correlated with high tumor burden (which we defined as stages III and IV), along with age and sex. The biomarkers showed higher AUC in predicting the severity of tumor burden compared with conventional tumor markers. In addition, the expression of PPL, miR301a, and miR140-5p significantly increased in the prospectively collected saliva of far-advanced gastric cancer (stage III and IV) patients at post-operative day 5 compared with day 0.

Conventional tumor markers CEA, CA19-9, and CA72-4 do not allow for the diagnosis of gastric cancer with adequate sensitivity and specificity; their use is limited to prognosis and follow-up recommendations [12]. Therefore, identifying conventional biomarkers that are noninvasive, highly specific, capable of early detection, and aiding treatment choice is urgent [13]. The use of liquid biopsies, which are samples of body fluid that may contain genetic material from a tumor, including blood, urine, saliva, or cerebrospinal fluid, may address some of these challenges [14].

Therefore, we performed multivariate analysis to evaluate which of the three candidate biomarkers reflected tumor burden. PPL performed better than the other two candidates, with a *p*-value less than 0.05 and an odds ratio per unit less than 1.00 in multivariate analysis. This indicates that the expression level of PPL decreases significantly when the tumor load increases, such as in pathological stages III and IV. In this study, the AUC was 89.2% in the multivariable model, including sex, age, and three biomarkers, in predicting the tumor burden (stages III and IV). This was higher than the AUC value of the multivariable model with three conventional tumor markers (70.5%). The use of salivary biomarkers may be a less invasive and more accurate method for risk stratification of patients before surgery than the evaluation of tumor markers through blood sampling.

The biological mechanisms underlying the development of discriminatory biomarkers in saliva from a tumor distal to the oral cavity have been established in previous studies. Mediators from distal tumors can alter salivary gland transcription factor activities responsible for the majority of gene and protein expression changes detected in saliva [5,15]. Tumor-derived exosomes, which are durable, cell-specific lipid microvesicles (30–200 nm) that migrate systemically through the vasculature of the body to facilitate intercellular communication [16,17], were the key mediators [18,19].

A previous study discovered and validated exRNA biomarkers in saliva with credible clinical performance for screening gastric cancer [8]. This study showed that three mRNAs (SPINK7, PPL, and SEMA4B) and two miRNAs (miR140-5p and miR301a) were significantly downregulated in gastric cancer patients. However, in our study, not all biomarkers were decreased before surgery, as expected. We assume that this result may have been influenced not only by tumor characteristics but also by patient factors, such as underlying systemic diseases and medications.

To eliminate the effects of individual differences on the results of salivary exRNA analysis, we employed an experimental strategy using pre- and post-operative paired salivary samples from the same cancer patients in this study. Among the five biomarkers, only PPL, miR140-5p, and miR310a were significantly increased five days after surgery in stage III and IV gastric cancer patients. The change in the three biomarkers during the immediate perioperative period might indicate that these are more sensitive biomarkers to gastric cancer and reflect a quicker response to treatment than the other markers. However, the kinetics and metabolism of the mediators from gastric cancer have not yet been clearly elucidated. In addition, surgical stress and changes in metabolism (catabolism) after surgery might influence the mediators and transcription of the salivary biomarkers. Previous studies demonstrated a significant decrease in the expression level of gastric cancer–released circulating miRNA in plasma one month after surgery [20,21]. Thus, future studies should collect post-operative salivary samples approximately one month after surgery to evaluate the change in salivary biomarkers.

Although our results are promising, our study has several limitations: (1) This study is a prospective, single-institution pilot study. As the sample size is small, validations in large cohorts are necessary; (2) There was no appropriate control group (healthy individuals). The inclusion of healthy controls would provide additional comparative value. However, this current study focused on evaluating the potential of salivary biomarkers to reflect tumor burden and surgical response in gastric cancer patients. To minimize inter-individual variability, we employed a pre-operative vs. post-operative paired analysis design; (3) There was no long-term regular follow-up of the expression levels of the salivary biomarkers; (4) Knowledge is limited on kinetics and mechanisms of gastric cancer–released factors that affect the transcription of salivary exRNA; (5) We did not compare tissue or blood-level RNA and protein expression data.

In conclusion, salivary extracellular RNAs may be a novel marker for gastric cancer, not only for detecting or screening cancer but also for estimating tumor burden before surgery and monitoring treatment response (especially after curative gastrectomy). Further prospective clinical trials using these biomarkers should be carried out to define their usefulness for potential applications.

## Figures and Tables

**Table 1:** Clinicopathologic characteristics of enrolled patients (*n* = 50).

Characteristic	No. of Patients (%)
Age, years	
<60	27 (54%)
≥60	23 (46%)
Sex	
Female	17 (34%)
Male	33 (66%)
*H. pylori* infection	
No	13 (26%)
Yes	20 (40%)
Unknown	17 (34%)
Histologic type	
Differentiated	22 (44%)
Undifferentiated	28 (56%)
Lauren type	
Intestinal	24 (48%)
Diffuse	20 (40%)
Mixed/indeterminate	6 (12%)
Size	
<6 cm	29 (58%)
≥6 cm	21 (42%)
Pathologic stage	
I	8 (16%)
II	16 (32%)
III	23 (46%)
IV	3 (6%)
Lymphatic invasion	
No	19 (38%)
Yes	31 (62%)

**Table 2:** Pre-operative expression levels of serum-derived tumor markers (CEA, CA19-9, and CA72-4) and salivary extracellular RNA biomarkers.

Internal Validation	Tumor Marker */Biomarker	Stage I/II		Stage III/IV		*p*-Value
		No. of Patients		No. of Patients		
N/A	CEA *	24	15.1 ± 13.2	26	1.5 ± 0.2	0.132
	CA19-9 *	24	18.3 ± 6.2	26	9.1 ± 1.4	0.954
	CA72-4 *	24	5.0 ± 1.8	26	3.1 ± 0.7	0.466
						
GAPDH	SPINK7	22	−4.8 ± 2.1	22	−4.9 ± 2.0	0.933
	SEMA4B	16	0.0 ± 1.7	11	0.3 ± 1.4	0.548
	PPL	22	−6.2 ± 2.4	20	−7.9 ± 2.4	0.025
						
ACTB	SPINK7	21	5.4 ± 2.0	25	5.8 ± 2.3	0.457
	SEMA4B	16	10.5 ± 0.9	12	11.9 ± 1.6	0.012
	PPL	21	4.1 ± 1.7	23	3.2 ± 1.7	0.075
						
U6	miR140-5p	23	−0.3 ± 1.2	23	0.1 ± 1.3	0.269
	miR301a	18	6.3 ± 1.5	21	5.9 ± 1.5	0.497
						
miR197	miR140-5p	24	−1.3 ± 0.8	24	−0.8 ± 0.7	0.036
	miR301a	18	5.2 ± 1.0	21	5.0 ± 1.1	0.531

Summary statistics are presented as mean ± standard deviation except for tumor markers *, which are presented as mean ± standard error. *p*-value was calculated using independent *t*-test (biomarkers) and Mann–Whitney U test (tumor markers *) to test the difference between pathologic stage I/II and stage III/IV.

**Table 3:** Univariate and multivariate predictive models for classification of pathologic stages III and IV using clinicopathologic factors and salivary extracellular RNA biomarkers.

Variable	N	Event * (%) (*n* = 26)	Univariable Model		Multivariable Model ^a^ (AUC = 89.2%)		Multivariable Model ^b-1^ (AUC = 75.4%)		Multivariable Model ^b-2^ (AUC = 82.5%)		Multivariable Model ^b-3^ (AUC = 75.5%)	
			OR (95% CI)	*p*-Value	OR (95% CI)	*p*-Value	OR (95% CI)	*p*-Value	OR (95% CI)	*p*-Value	OR (95% CI)	*p*-Value
Age, years												
<60	27	15 (55.6)	1		1		1		1		1	
≥60	23	11 (47.8)	0.73 (0.24, 2.24)	0.586	1.07 (0.15, 7.64)	0.946	0.73 (0.2, 2.64)	0.630	0.72 (0.14, 3.85)	0.702	0.85 (0.24, 2.99)	0.794
Sex												
Female	17	12 (70.6)	1		1		1		1		1	
Male	33	14 (42.4)	0.31 (0.09, 1.07)	0.064	0.2 (0.03, 1.6)	0.129	0.3 (0.07, 1.25)	0.098	0.22 (0.04, 1.39)	0.107	0.27 (0.07, 1.01)	0.052
Biomarkers (per 1 unit)												
PPL/GAPDH	47	24 (51.1%)	0.74 (0.57, 0.96)	0.025	0.83 (0.54, 1.26)	0.378	0.74 (0.56, 0.96)	0.026				
SEMA4B/b-actin	37	20 (54.1%)	2.75 (1.31, 5.78)	0.008	2.54 (0.93, 6.94)	0.070			3.13 (1.29, 7.59)	0.012		
miR140/miR197	49	25 (51.0%)	2.47 (1.08, 5.64)	0.032	3.62 (0.81, 16.24)	0.093					2.52 (1.06, 5.98)	0.036

* Event is presented as Stages III + IV. Multivariable models were adjusted for age and sex. ^a^ Multivariable model combining clinical characteristics (age, sex) and biomarkers (PPL/GAPDH, SEMA4B/b-actin, and miR140/miR197). ^b-1^ Multivariable model combining clinical characteristics (age, sex) and biomarker (PPL/GAPDH). ^b-2^ Multivariable model combining clinical characteristics (age, sex) and biomarker (SEMA4B/b-actin). ^b-3^ Multivariable model combining clinical characteristics (age, sex) and biomarker (miR140/miR197).

**Table 4:** Univariate and multivariate predictive models for classification of pathologic stages III and IV using clinicopathologic factors and tumor markers (CEA, CA19-9, CA72-4).

Variable	Total (*n* = 50)	Event * (%) (*n* = 26)	Univariable Model		Multivariable Model ^c^ (AUC = 70.5%)		Multivariable Model ^d−1^ (AUC = 67.2%)		Multivariable Model ^d−2^ (AUC = 66.2%)		Multivariable Model ^d−3^ (AUC = 67.4%)	
			OR (95% CI)	*p*-Value	OR (95% CI)	*p*-Value	OR (95% CI)	*p*-Value	OR (95% CI)	*p*-Value	OR (95% CI)	*p*-Value
Age, years												
<60	27	15 (55.6)	1		1		1		1		1	
≥60	23	11 (47.8)	0.73 (0.24, 2.24)	0.586	0.96 (0.28, 3.27)	0.946	0.91 (0.28, 3.00)	0.878	0.83 (0.26, 2.73)	0.765	0.78 (0.24, 2.50)	0.672
Sex												
Female	17	12 (70.6)	1		1		1		1		1	
Male	33	14 (42.4)	0.31 (0.09, 1.07)	0.064	0.41 (0.11, 1.55)	0.190	0.37 (0.10, 1.35)	0.132	0.33 (0.09, 1.17)	0.085	0.32 (0.09, 1.14)	0.080
Tumor markers												
CEA (ng/mL)	50	26 (52.0%)	0.65 (0.34, 1.25)	0.200	0.72 (0.35, 1.47)	0.364	0.74 (0.37, 1.48)	0.397				
CA19-9 (U/mL)	50	26 (52.0%)	0.97 (0.93, 1.02)	0.200	0.98 (0.94, 1.02)	0.224			0.97 (0.93, 1.02)	0.257		
CA72-4 (U/mL)	50	26 (52.0%)	0.95 (0.85, 1.06)	0.366	0.96 (0.87, 1.06)	0.433					0.96 (0.85, 1.07)	0.431

* Event is presented as Stage III + IV. Multivariable models were adjusted for age and sex. ^c^ Multivariable model combining clinical characteristics (age, sex) and tumor markers (CEA, CA19-9 and CA72-4). ^d-1^ Multivariable model combining clinical characteristics (age, sex) and tumor marker (CEA). ^d-2^ Multivariable model combining clinical characteristics (age, sex) and tumor marker (CA19-9). ^d-3^ Multivariable model combining clinical characteristics (age, sex) and tumor marker (CA72-4).

**Table 5:** Differences in expression levels of salivary extracellular RNA biomarkers before and after gastrectomy.

Internal Validation	No. of Patients	Biomarker	Pre-Operative (Day 0)	Post-Operative (Day 5)	Day 5–Day 0	*p*-Value
						
GAPDH	44	SPINK7	−4.8 ± 2.0	−5.2 ± 2.5	−0.3 ± 2.6	0.398
	27	SEMA4B	0.1 ± 1.6	0.2 ± 1.8	0.1 ± 2.4	0.843
	42	PPL	−7.0 ± 2.5	−6.3 ± 2.6	0.8 ± 3.1	0.113
						
ACTB	46	SPINK7	5.6 ± 2.2	5.4 ± 2.7	−0.2 ± 3.4	0.640
	28	SEMA4B	11.1 ± 1.4	11.2 ± 1.3	0.1 ± 1.6	0.650
	44	PPL	3.7 ± 1.7	3.9 ± 2.0	0.3 ± 1.9	0.331
						
U6	46	miR140-5p	−0.1 ± 1.3	0.1 ± 1.7	0.2 ± 1.8	0.453
	39	miR301a	6.1 ± 1.5	6.3 ± 1.7	0.3 ± 1.8	0.399
						
miR197	48	miR140-5p	−1.0 ± 0.8	−0.6 ± 0.9	0.4 ± 0.9	0.002
	39	miR301a	5.1 ± 1.0	5.8 ± 1.2	0.7 ± 1.5	0.006

Summary statistics are presented as mean ± standard deviation. *p*-value was calculated using paired *t*-test to test the difference in change (Day 5–Day 0) between groups.

**Table 6:** Differences in expression levels of salivary extracellular RNA biomarkers before and after gastrectomy in far-advanced gastric cancer (pathologic stage III and IV) patients.

Internal Validation	No. of Patients	Biomarkers	Pre-Operative (Day 0, D0)	Post-Operative (Day 5, D5)	Day 5–Day 0	*p*-Value
						
GAPDH	22	SPINK7	−4.9 ± 2.0	−4.7 ± 2.6	0.2 ± 2.7	0.712
	11	SEMA4B	0.3 ± 1.4	0.2 ± 1.6	−0.2 ± 2.0	0.780
	20	PPL	−7.9 ± 2.4	−6.1 ± 2.5	1.8 ± 3.2	0.019
						
ACTB	25	SPINK7	5.8 ± 2.3	6.0 ± 2.9	0.2 ± 3.7	0.788
	12	SEMA4B	11.9 ± 1.6	11.5 ± 1.2	−0.4 ± 2.0	0.518
	23	PPL	3.2 ± 1.7	3.9 ± 1.9	0.7 ± 2.0	0.104
						
U6	23	miR140-5p	0.1 ± 1.3	0.1 ± 1.9	−0.1 ± 2.0	0.888
	21	miR301a	5.9 ± 1.5	6.1 ± 1.6	0.1 ± 1.7	0.740
						
miR197	24	miR140-5p	−0.8 ± 0.7	−0.4 ± 1.0	0.4 ± 0.9	0.042
	21	miR301a	5.0 ± 1.1	5.8 ± 1.3	0.8 ± 1.6	0.039

Summary statistics are presented as mean ± standard deviation. *p*-value was calculated using paired *t*-test to test the difference in change (Day 5–Day 0) between groups.

## Data Availability

The data that support the findings of this study are available from the corresponding author upon reasonable request.

## Supplementary Materials

The following Supporting Information can be downloaded at: https://www.mdpi.com/article/10.3390/jcm14103596/s1, Table S1: Univariate and multivariate predictive models for classification of pathologic stage III and IV using clinicopathologic factors and salivary extracellular RNA biomarkers; Table S2: Expression of miRNA (miR140, miR301) based on miR197; Figure S1: ROC curves for the three biomarkers.

## Abbreviations

KNCSP	Korean National Cancer Screening Program
ExRNA	extracellular RNA
AUC	area under curve
ROC	receiver operating characteristic
RT-qPCR	real-time polymerase chain reaction
