# Supplementary material for: Diagnostic Significance in Estimating Tumor Burden Using Extracellular Salivary Biomarkers in Gastric Cancer Patients
Source: J Clin Med. 2025 May 21;14(10):3596. doi: 10.3390/jcm14103596 (PMC12112491; doi:10.3390/jcm14103596)
Supplement: Supplementary file 1 [file jcm-14-03596-s001.zip › jcm-3551438-supplementary.pdf]

Supplementary Table S1. Univariate and multivariate predictive models for classification of pathologic stage III and IV using clinicopathologic factors and salivary extracellular RNA biomarkers

Variable	Total (n = 50)	Event* (%) (n = 26)	Univariable model		Multivariable model[a] (AUC = 92.0%)		Multivariable model[b-1] (AUC = 84.1%)		Multivariable model[b-2] (AUC = 87.9%)		Multivariable model[b-3] (AUC = 81.7%)	
			OR (95% CI)	*p*-value	OR (95% CI)	*p*-value	OR (95% CI)	*p*-value	OR (95% CI)	*p*-value	OR (95% CI)	*p*-value
Age, years												
<60	27	15 (55.6%)	1		1		1		1		1	
≥60	23	11 (47.8%)	0.73 (0.24, 2.24)	0.586	0.81 (0.13, 5.00)	0.818	0.60 (0.13, 2.71)	0.506	0.68 (0.12, 3.86)	0.660	0.65 (0.17, 2.58)	0.544
Sex												
Female	17	12 (70.6%)	1		1		1		1		1	
Male	33	14 (42.4%)	0.31 (0.09, 1.07)	0.064	0.41 (0.04, 4.58)	0.466	0.88 (0.14, 5.41)	0.887	0.28 (0.03, 2.64)	0.268	0.63 (0.13, 3.11)	0.573
H. pylori infection												
No	13	4 (30.8%)	1	(0.221)	1		1		1		1	
Yes	20	12 (60.0%)	3.37 (0.77, 14.81)	0.107	1.67 (0.17, 16.62)	0.660	0.96 (0.14, 6.60)	0.964	3.46 (0.41, 29.15)	0.254	1.91 (0.35, 10.29)	0.454
Unknown	17	10 (58.8%)	3.21 (0.70, 14.74)	0.133	2.52 (0.23, 28.12)	0.453	1.52 (0.20, 11.60)	0.688	4.61 (0.49, 43.55)	0.182	2.63 (0.43, 16.05)	0.295
Histologic type												
Differentiated	22	7 (31.8%)	1		1		1		1		1	
Undifferentiated	28	19 (67.9%)	4.52 (1.37, 14.98)	0.014	0.51 (0.01, 32.34)	0.753	6.90 (0.15, 327.77)	0.327	0.54 (0.01, 31.76)	0.766	7.23 (0.19, 280.83)	0.290
Lauren type												
Intestinal	24	10 (41.7%)	1	(0.118)	1		1		1		1	
Diffuse	20	14 (70.0%)	3.27 (0.93, 11.45)	0.064	3.19 (0.07, 144.51)	0.551	0.70 (0.02, 28.52)	0.849	2.86 (0.05, 175.27)	0.616	0.35 (0.01, 11.53)	0.559
Mixed/indeterminate	6	2 (33.3%)	0.70 (0.11, 4.59)	0.710	3.25 (0.02, 653.21)	0.664	0.40 (0, 85.91)	0.740	1.76 (0.02, 152.22)	0.804	0.12 (0, 6.91)	0.302
Size												
<6 cm	29	14 (48.3%)	1		1		1		1		1	
≥6 cm	21	12 (57.1%)	1.43 (0.46, 4.42)	0.536	2.77 (0.39, 19.50)	0.306	3.30 (0.64, 17.19)	0.156	2.09 (0.32, 13.69)	0.442	1.22 (0.33, 4.5)	0.763
Biomarkers (per 1 units)												
PPL/GAPDH	47	24 (51.1%)	0.74 (0.57, 0.96)	0.025	0.79 (0.47, 1.30)	0.350	0.66 (0.44, 0.98)	0.041				
SEMA4B/b-actin	37	20 (54.1%)	2.75 (1.31, 5.78)	0.008	1.74 (0.73, 4.16)	0.210			2.30 (1.00, 5.28)	0.051		
miR140/miR197	49	25 (51.0%)	2.47 (1.08, 5.64)	0.032	1.86 (0.46, 7.49)	0.382					1.6 (0.70, 3.66)	0.265

*Event is presented as Stage III+IV. *AUC* area under the curve, *OR* odds ratio, *CI* confidence interval. Multivariable model adjusted for age, sex, *H. pylori* infection, histologic type, Lauren type, and size. In multivariable model, Firth's penalized likelihood approach was applied due to the small sample size

Supplementary Table S2. Expression of miRNA (miR140, miR301) based on miR197

Characteristics		miR140										miR301									
	N	*n*	Preop (D0)	*p*-value[a]	*n*	Postop (D5)	*p*-value[a]	*n*	(D5-D0)	*p*-value[b]	*p*-value[c]	*n*	Preop (D0)	*p*-value[a]	*n*	Postop (D5)	*p*-value[a]	*n*	(D5-D0)	*p*-value[b]	*p*-value[c]
Total		48	-1.03 ± 0.81		48	-0.61 ± 0.92		48	0.42 ± 0.89	0.0021		39	5.1 ± 1.02		39	5.8 ± 1.18		39	0.7 ± 1.5	0.0056	
Age																					
<60	27	26	-0.94 ± 0.78	0.4101	26	-0.65 ± 1.11	0.7357	26	0.29 ± 0.87	0.1049	0.2759	20	5.07 ± 1.04	0.8412	20	5.82 ± 1.37	0.9292	20	0.75 ± 1.61	0.0493	0.8362
≥60	23	22	-1.13 ± 0.85		22	-0.56 ± 0.67		22	0.57 ± 0.9	0.0073		19	5.13 ± 1.03		19	5.79 ± 0.99		19	0.65 ± 1.42	0.0606	
Sex																					
Female	17	17	-0.97 ± 0.75	0.7054	17	-0.58 ± 0.58	0.8574	17	0.39 ± 0.76	0.0516	0.8532	15	5.18 ± 1.02	0.7120	15	5.96 ± 1.24	0.5225	15	0.78 ± 1.14	0.0187	0.8012
Male	33	31	-1.06 ± 0.85		31	-0.62 ± 1.08		31	0.44 ± 0.97	0.0176		24	5.05 ± 1.04		24	5.71 ± 1.16		24	0.66 ± 1.71	0.0726	
H.pylori infection*																					
No	13	13	-1.33 ± 0.69	0.1787	13	-1.03 ± 0.89	0.0102	13	0.3 ± 0.91	0.2615	0.3248	12	5.64 ± 0.77	0.0332	12	6.08 ± 1.06	0.5100	12	0.44 ± 1.43	0.3063	0.7279
Yes	20	20	-0.8 ± 0.76		20	-0.15 ± 0.97		20	0.65 ± 0.88	0.0039		17	5.06 ± 1.14		17	5.79 ± 1.37		17	0.74 ± 1.71	0.0946	
Unknown	17	15	-1.08 ± 0.92		15	-0.85 ± 0.62		15	0.22 ± 0.88	0.3470		10	4.52 ± 0.79		10	5.48 ± 0.99		10	0.96 ± 1.27	0.0400	
Histologic type																					
Differentiated	22	21	-1.2 ± 0.86	0.1897	21	-0.7 ± 0.75	0.5721	21	0.51 ± 0.78	0.0074	0.5517	17	5.2 ± 1.23	0.5824	17	6.21 ± 1.21	0.0587	17	1.01 ± 1.56	0.0172	0.2758
Undifferentiated	28	27	-0.89 ± 0.75		27	-0.54 ± 1.05		27	0.35 ± 0.98	0.0739		22	5.02 ± 0.85		22	5.49 ± 1.09		22	0.47 ± 1.44	0.1400	
Lauren type*																					
Intestinal	24	23	-1.12 ± 0.86	0.5720	23	-0.56 ± 0.71	0.2659	23	0.56 ± 0.73	0.0013	0.3600	19	5.23 ± 1.2	0.7395	19	6.14 ± 1.19	0.1995	19	0.92 ± 1.5	0.0159	0.6888
Diffuse	20	19	-0.87 ± 0.83		19	-0.49 ± 1.17		19	0.38 ± 1.09	0.1445		15	5.01 ± 0.87		15	5.55 ± 1.2		15	0.54 ± 1.64	0.2210	
Mixed/Indeterminated	6	6	-1.16 ± 0.54		6	-1.18 ± 0.65		6	-0.02 ± 0.65	0.9419		5	4.88 ± 0.77		5	5.27 ± 0.85		5	0.39 ± 1.19	0.5053	
Size																					
<6cm	29	29	-0.98 ± 0.81	0.6341	29	-0.54 ± 1.05	0.5296	29	0.44 ± 0.93	0.0165	0.8261	24	5.14 ± 1.11	0.7533	24	5.7 ± 1.14	0.5045	24	0.56 ± 1.3	0.0451	0.4583
≥6cm	21	19	-1.1 ± 0.83		19	-0.71 ± 0.71		19	0.38 ± 0.84	0.0635		15	5.03 ± 0.91		15	5.97 ± 1.28		15	0.93 ± 1.8	0.0644	
Pathologic stage																					
I+II	24	24	-1.27 ± 0.84	0.0359	24	-0.84 ± 0.75	0.0860	24	0.43 ± 0.88	0.0240	0.9161	18	5.21 ± 0.95	0.5314	18	5.82 ± 1.07	0.9359	18	0.61 ± 1.37	0.0759	0.7172
III+IV	26	24	-0.79 ± 0.71		24	-0.38 ± 1.03		24	0.4 ± 0.92	0.0423		21	5 ± 1.09		21	5.79 ± 1.3		21	0.79 ± 1.63	0.0392	
Lymphatic invasion																					
No	19	19	-1.22 ± 0.74	0.1891	19	-0.71 ± 0.54	0.4749	19	0.5 ± 0.82	0.0149	0.5939	14	5.02 ± 0.96	0.7335	14	5.83 ± 1.12	0.9334	14	0.8 ± 1.53	0.0718	0.7658
Yes	31	29	-0.9 ± 0.84		29	-0.54 ± 1.11		29	0.36 ± 0.94	0.0484		25	5.14 ± 1.07		25	5.79 ± 1.24		25	0.65 ± 1.51	0.0417	
Venous invasion																					
No	41	40	-1.07 ± 0.79	0.3979	40	-0.65 ± 0.92	0.5315	40	0.42 ± 0.93	0.0062	0.9075	31	5.21 ± 1.02	0.1704	31	5.66 ± 1.22	0.1364	31	0.45 ± 1.44	0.0955	0.0319
Yes	9	8	-0.8 ± .91		8	-0.42 ± 0.99		8	0.38 ± 0.71	0.1675		8	4.65 ± 0.95		8	6.36 ± 0.9		8	1.71 ± 1.36	0.0093	
Perineural invasion																					

No	18	18 -1.13 ± 0.7	0.5130	18 -0.68 ± 0.59	0.6221	18 0.44 ± 0.86	0.0426	0.8821	14 5.11 ± 1.01	0.9432	14 5.87 ± 1.01	0.7935	14 0.76 ± 1.58	0.0970	0.8746
Yes	32	30 -0.97 ± 0.87		30 -0.56 ± 1.08		30 0.4 ± 0.92	0.0233		25 5.09 ± 1.05		25 5.77 ± 1.29		25 0.68 ± 1.48	0.0320	

Summary statistics were presented as mean ± standard deviation. *P* value[a] was calculated using independent t-test. *P* value[b] was calculated using paired t-test. [*]*P* value[a] was calculated using one-way ANOVA test. *P* value[c] was calculated using independent t-test or one-way ANOVA test to confirm the difference of change (D5-D0) between groups.

Supplementary Figure S1. ROC curves for the three biomarkers

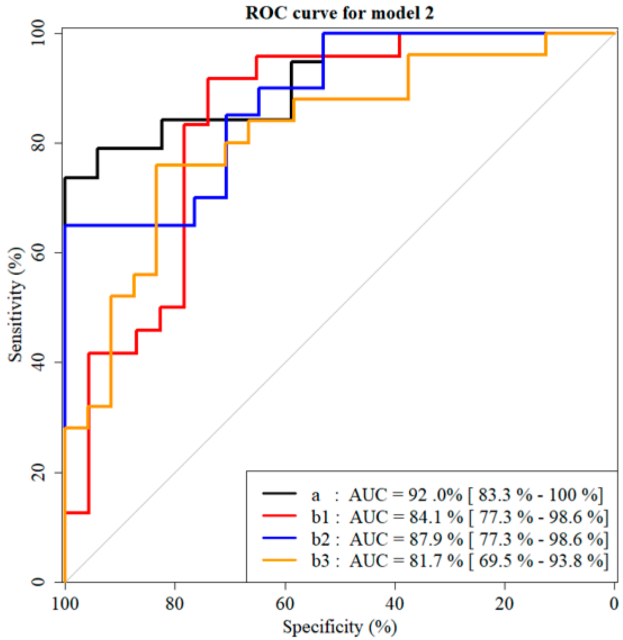

			Probability	Sensitivity	Specificity
Multivariable model combining clinical characteristics and biomarkers	a	Age + Sex + H.pylori infection + Histologic type + Lauren type + Size + PPL/GAPDH + SEMA4B/b-actin + miR140/miR197	67.9%	73.7%	100.0%
	b-1	Age + Sex + H.pylori infection + Histologic type + Lauren type + Size + PPL/GAPDH	42.1%	91.7%	73.9%
	b-2	Age + Sex + H.pylori infection + Histologic type + Lauren type + Size + SEMA4B/b-actin	67.6%	65.0%	100.0%
	b-3	Age + Sex + H.pylori infection + Histologic type + Lauren type + Size + miR140/miR197	52.1%	76.0%	83.3%
